# Multibehavioural Interventions with a Focus on Specific Energy Balance-Related Behaviours Can Affect Diet Quality in Preschoolers from Six European Countries: The ToyBox-Study

**DOI:** 10.3390/nu9050479

**Published:** 2017-05-10

**Authors:** An-Sofie Pinket, Marieke De Craemer, Inge Huybrechts, Ilse De Bourdeaudhuij, Benedicte Deforche, Greet Cardon, Odysseas Androutsos, Berthold Koletzko, Luis A. Moreno, Piotr Socha, Violeta Iotova, Yannis Manios, Wendy Van Lippevelde

**Affiliations:** 1 Department of Public Health, Ghent University, 9000 Ghent, Belgium; Benedicte.Deforche@UGent.be (B.D.); Wendy.VanLippevelde@UGent.be (W.V.L.); 2 Department of Movement and Sports Sciences, Ghent University, 9000 Ghent, Belgium; Marieke.DeCraemer@UGent.be (M.D.C.); Ilse.DeBourdeaudhuij@UGent.be (I.D.B.); Greet.Cardon@UGent.be (G.C.); 3 International Agency for Research on Cancer (IARC/WHO), 69000 Lyon, France; Inge.Huybrechts@UGent.be; 4 Department of Human Biometry and Biomechanics, Vrije Universiteit Brussel, 1000 Brussels, Belgium; 5 Department of Nutrition and Dietetics, School of Health Science and Education, Harokopio University, 17671 Athens, Greece; oandrou@hua.gr (O.A.); manios.toybox@hua.gr (Y.M.); 6 Ludwig-Maximilians-University of Munich, Dr. von Hauner Children’s Hospital, 80337 München, Germany; Berthold.Koletzko@med.uni-muenchen.de; 7 GENUD Research Group, University of Zaragoza, 50009 Zaragoza, Spain; lmoreno@unizar.es; 8 The Children’s Memorial Health Institute, 04-730 Warsaw, Poland; p.socha@czd.pl; 9 Department of Pediatrics, Medical University of Varna, 9002 Varna, Bulgaria; iotova_v@yahoo.com

**Keywords:** young children, diet quality, ToyBox-study, multibehavioural intervention, socioeconomic status

## Abstract

The present study aimed to examine whether a multibehavioural intervention with a focus on specific energy balance-related behaviours can affect total diet quality and its four subcomponents in European preschoolers and to investigate if these intervention effects differed by socioeconomic status (SES). Parents/caregivers of 3.5 to 5.5 year-olds (*n* = 4968) recruited through kindergartens in six European countries within the ToyBox-study completed questionnaires on socio-demographics and a food frequency questionnaire on their preschoolers’ diet. To assess intervention effects and differences by SES, multilevel repeated measures analyses were conducted. In contrast to no significant difference in total diet quality, in both the intervention and control group, the dietary quality and dietary equilibrium increased, with a larger increase in the intervention group (mean difference _quality_: +3.4%; mean difference _equilibrium_: +0.9%) compared to the control group (_quality_: +1.5%; _equilibrium_: +0.2%). SES was not a significant moderator for intervention effects on total diet quality, nor for the four subcomponents. This study indicates that multibehavioural interventions with a focus on specific energy balance-related behaviours in preschoolers not only affect those targeted behaviours, but can also have more generalized effects. The ToyBox-intervention effects were similar for both lower and high SES preschoolers.

## 1. Introduction

The dietary intake and nutritional status of European (preschool) children is found to be rather poor [1,2,3,4]. As dietary habits are being formed at a young age and track into later childhood and adulthood, it is important to intervene early in life and focus on changing unhealthy dietary behaviours already at preschool age [5,6]. Until now, most intervention studies focusing on dietary behaviours in preschool aged children evaluated specific dietary habits, i.e., breakfast frequency, consumption of fruit and vegetables, soft drinks, body mass index (BMI) or BMI z-scores or total energy intake as outcomes, rather than overall diet quality [7,8,9,10,11]. However, by using a diet quality index to evaluate the food habits of preschool children, the complex and multidimensional nature of eating patterns is covered [12]. This means that using a diet quality index can help to get an overview of the total diet quality of preschoolers in line with holistic dietary guidelines. By focusing on specific dietary behaviours, only a part of the total diet quality is evaluated while the holistic evaluation is missing.

A Diet Quality Index (DQI) specifically for preschoolers consisting of four subcomponents, dietary diversity, dietary quality, dietary equilibrium, and the meal index, was developed and validated by Huybrechts and colleagues. These subcomponents are key components of a balanced diet in preschool children. Dietary diversity stresses the importance of food intake variety to meet the macro- and micronutrient needs [13,14,15,16,17]. The dietary quality subcomponent states that high nutritious food items should be recommended and energy-dense, low-nutritious food items should be discouraged [13,14,15,18]. The dietary equilibrium score is composed of two subcomponents: (1) the adequacy scores indicate to what extent preschoolers meet the minimum food recommendations; while (2) the excess/moderation score indicates to what extent preschoolers exceed maximum recommendations [13,14,15]. The last subcomponent of total diet quality is the meal index, stressing the importance of breakfast, lunch, and dinner every day [19]. The total DQI is calculated by summing the four index components. The maximum score for total diet quality and each subcomponent is 100%. Higher scores indicate better scores on total diet quality or on the subcomponent than lower scores [13,14,15]. Studies investigating the overall diet quality of preschool children based on the Diet Quality Index developed by Huybrechts and colleagues are scarce. One Flemish study found rather low scores on overall diet quality based on this index [14]. In addition, the index has been used in a large-scale European study among adolescents showing significant positive associations with essential nutritional biomarkers and negative associations with rather unhealthy/detrimental biomarker indicators such as trans fatty acids [20,21].

Until now, it has been unclear whether multibehavioural interventions focusing on specific energy balance-related behaviours in preschool children, such as increasing consumption of fruit and vegetables and reducing soft drink consumption, also have an effect on the overall diet quality, by, for example, raising awareness for an overall healthy lifestyle. Studies evaluating the effect of a multibehavioural intervention, i.e., an intervention targeting more than one health behaviour, on overall diet quality are currently missing in preschool children.

The ToyBox-study (multifactorial evidence-based approach using behavioural models in understanding and promoting fun, healthy food, play, and policy for the prevention of obesity in early childhood) was a European Union-funded large-scale study in 3.5–5.5 year-old preschoolers and their families from six European countries: Belgium, Bulgaria, Germany, Greece, Poland, and Spain [22]. Within the ToyBox-study, a kindergarten-based, family-involved intervention was developed, implemented, and tested. The ToyBox-intervention targeted four key behaviours related to early childhood obesity: water consumption, snacking behaviour, physical activity, and sedentary behaviour. Previous publications on the ToyBox-intervention showed promising results on different health behaviours, with a larger decrease of prepacked fruit juice consumption and a larger increase of vigorous and moderate-to-vigorous physical activity in preschoolers of the intervention group compared to preschoolers of the control group [23,24,25]. In addition, preschool children included in the ToyBox-study were found to score low on total diet quality and three subcomponents at baseline [13]. The majority of preschoolers lack variety in their diet, i.e., dietary diversity, consume energy-dense low-nutritious food items such as sweet snacks instead of highly nutritious food items such as fresh fruit, i.e., dietary quality, and do not reach minimum food recommendations such as for vegetable intake, while they exceed maximum recommendations such as for sweet snacks, i.e., dietary equilibrium [13]. In addition to the already low scores on diet quality, lower socioeconomic status (SES) preschoolers were found to have even lower scores on total diet quality and all subcomponents than their high SES peers [13]. Preventing the further development of these inequalities early in life is vital to tackle socioeconomic inequalities in health.

Therefore, the first purpose of this study was to examine whether the ToyBox-intervention, a multibehavioural intervention with a focus on specific energy balance-related behaviours, can affect total diet quality and the four subcomponents of diet quality developed by Huybrechts et al. [14,15] in European preschoolers. The second aim of this study was to investigate if the intervention effects on diet quality differed by socioeconomic status, as only few studies have explored the differential effect of interventions by SES, especially in preschoolers [26].

## 2. Materials and Methods

### 2.1. Study Protocol

Within the ToyBox-study, an intervention with a randomized cluster design was developed following the PRECEDE-PROCEED model and the Intervention Mapping protocol [27]. The intervention consisted of a pre- and post-test design with intervention and control kindergartens in all six European countries. The ToyBox-study (www.toybox-study.eu) is registered with the clinical trials registry (clinical_trials.gov, ID: NCT02116296) and was approved by Ethical Committees in all six European countries, in line with national regulations (i.e., the Ethical Committee of Ghent University Hospital (Ghent, Belgium), Committee for the Ethics of the Scientific Studies (KENI) at the Medical University of Varna (Varna, Bulgaria), Ethikkommission der Ludwig-Maximilians-Universität München (München, Germany), the Ethics Committee of Harokopio University of Athens (Athens, Greece), Ethical Committee of Children’s Memorial Health Institute (Warsaw, Poland), and CEICA (Comité Ético de Investigación Clínica de Aragón (Zaragoza, Spain)) [22]. Parents/caregivers were asked for written informed consent for the participation of their preschool child in the study.

### 2.2. Sampling

The time plan of the ToyBox-intervention was designed so as to account for country-specific differences with regard to the opening and closing dates of the kindergartens and the duration and timing of national holidays. After recruitment of participants started in February 2012, baseline data was collected between May and June 2012 [22].

A minimum sample of 800 children and their families and 20 kindergartens per country, resulting in a total sample of 4800 children and their families and 120 kindergartens, was initially targeted. However, in order to account for an estimated dropout rate of about 30%, a minimum total number of about 6500 children and their families were aimed to be recruited in the six participating countries. Detailed power calculations are described elsewhere [22]. The preschool children and their families were recruited at kindergartens, daycare centres, or preschool settings, depending on the country regulations and legislation [22]. In order to avoid confusion for the reader, all these settings (kindergartens, daycare centres, preschool settings) will be referred to as “kindergartens” in this paper.

Kindergartens were recruited from different socio-demographic backgrounds within each of the provinces in the different countries. Lists of all municipalities that exist within the selected provinces were created with information on SES variables of the municipalities (i.e., average of education years of the population aged between 25 and 55 years or mean annual household income). Tertiles including three different socio-demographic groups were created based on the selected SES variables, and each country randomly selected approximately five municipalities per SES status (low, medium, and high SES). Then, kindergartens within these randomly chosen municipalities were randomly selected (with the exclusion of the lowest 20% of the kindergartens with the smallest number of pupils) [22]. The selected kindergartens were visited by researchers to inform the kindergarten staff about the ToyBox-study. After kindergartens agreed to participate in the study, preschoolers received an information letter to take home with information on the study for parents/caregivers. Subsequently, municipalities were randomly assigned to the intervention or the control condition (2:1) [22]. Kindergartens of the ToyBox-intervention group received the intervention, while kindergartens of the control group were asked to continue the normal kindergarten routine.

### 2.3. The ToyBox-Intervention

The ToyBox-intervention was implemented within the academic year 2012–2013 and the implementation was conducted at four levels. The first three levels were implemented in the kindergarten settings and the fourth level focused on changing the home environment through parents/caregivers. As mentioned previously, the ToyBox-intervention targeted four key behaviours related to early childhood obesity: water consumption, snacking behaviour, physical activity, and sedentary behaviour. During the first level of implementation, kindergarten teachers made environmental changes in the kindergartens. In this way, a supportive environment for all four key behaviours was created, for instance, by installing water stations and magic snack plates. In the second implementation level, kindergarten teachers promoted the four key behaviours on a regular basis, for instance by reminding preschoolers to drink water regularly and by arranging a daily break for the whole class to eat healthy snacks. In the third level of implementation, kindergarten teachers implemented fun classroom activities, such as kangaroo stories, experiments, games, and excursions, for a minimum of one hour per week. The ToyBox-intervention materials were provided to kindergarten teachers in a box. This box contained a teacher’s guide with general information on the ToyBox-intervention and the importance of the four key behaviours, a classroom activity guide for each key behaviour and a kangaroo hand puppet. The classroom activity guides consisted of three sections, matching the first three levels of implementation: setting environmental changes in the classroom, preschoolers implementing the actual behaviour, and teachers implementing fun classroom activities. Detailed information is described elsewhere [22]. The fourth level of implementation targeted parents/caregivers. Preschoolers received nine newsletters, eight tip-cards, and four posters to take home. The third level of each key behaviour was focused on for four weeks, followed by a repetition period in which each key behaviour was focused on for another two weeks.

Prior to the intervention, teachers were invited to two teacher training sessions in which researchers explained the ToyBox-intervention and provided detailed information on how to implement the materials. A third teacher training session was scheduled before the repetition period [28,29].

### 2.4. Measures

#### 2.4.1. Food and Beverage Intake

Parents/caregivers completed a semi-quantitative food frequency questionnaire (FFQ) for preschool children, in which they described preschoolers’ food and beverage intake over the last 12 months. The FFQ was developed based on a previously validated FFQ developed by Huybrechts et al. [30]. For each of the food (e.g., yogurt and cheese, fruit and vegetables, chocolate and desserts, cookies and pastry, cereals, bread, salty snacks, meat and fish, potatoes, rice and pasta, sugar, jam and other spreads, legumes) and beverage (e.g., plain water, tea, milk, sugared milk, fruit juices, soft drinks, light soft drinks) items, the frequency of consumption and portion size were asked. Response categories for frequency were: “never or less than once per month”, “1–3 days per month”, “1 day per week”, “2–4 days per week”, “5–6 days per week”, and “every day”. The response categories for portion size varied depending on the food item and parents/caregivers were asked to indicate the portion size category that best fitted the daily portion of their child. To facilitate the selection of portion sizes, a list of common standard measures was given as example, as well as colour images. Dietary data from the FFQ were converted to average daily intake values by multiplication of number of days per week and amount per day divided by 7 [30].

To avoid missing scores, the following encodings in the FFQ data were made, based on encoding of previous papers [13,30]. When no portion size was selected and the frequency category chosen was “never or less than once a week”, it was considered that the children did (almost) never consume the item and the portion size was recoded into ‘0’. When no portion size was selected but the frequency was not missing and not “never or less than once a week”, the median of the portion size of that item was given as the code on this item. Children with both a missing frequency and portion size on an item of the FFQ were considered as non-consumers and were recoded into ‘0’, both on frequency and portion size of that item. When the frequency was missing but a portion size was mentioned, the code on frequency was replaced by the median of the frequency of that item.

#### 2.4.2. Diet Quality Index (DQI)

The DQI for preschool children was developed based on the Flemish active food triangle and assesses the compliance of preschoolers with the Flemish food-based dietary guidelines [15,31]. The triangle recommends a daily intake for each food group (non-sugared beverages; bread, cereals, potatoes, and grains; vegetables; fruit; milk products and calcium-enriched soy products; meat, fish, eggs, and meat substitutes; fat and oils) in order to cover the varying nutrient needs in the population. The tip of the triangle is separated from the rest of the triangle and is called the ‘rest group’ including snacks and sugared beverages [1]. The DQI is based on Flemish guidelines, because there are no European guidelines yet. However, the Flemish recommendations are very similar to dietary guidelines in other countries, making these recommendations applicable for a European population of preschoolers [32].

To compute the total diet quality index (DQI) and the four subcomponents, all food and beverage items of the FFQ were used. The FFQ-based DQI score is a reasonable estimate of diet quality when compared with three-day diet records [14]. Detailed information on the calculation of total diet quality and the subcomponents can be found in Table 1 and is also described elsewhere [13,14,15].

#### 2.4.3. Socio-Economic Status and Other Socio-Demographic Variables

Socio-demographics, such as preschoolers’ sex and date of birth and parents’/caregivers’ SES, were reported in the parent questionnaire by preschoolers’ parents/caregivers. The educational level of the mother was then used as a SES indicator, since this parameter has been identified as an important indicator for SES [33]. The educational level was dichotomized into lower (14 or fewer years of education) and high (more than 14 years of education) SES, which distinguishes families with a mother who has completed medium or higher education (college or university training) from other families [34]. Date of birth and the date when the questionnaire was completed were used to compute preschoolers’ age. All questionnaires are available on the ToyBox-website (www.toybox-study.eu) and in the ToyBox supplement issue [35].

### 2.5. Statistical Analyses

Descriptive statistics were performed using IBM SPSS Statistics for Windows, version 21.0 (IBM Corp, Amonk, NY, USA). To assess the effectiveness of the intervention on total diet quality and the four subcomponents, multilevel repeated measures analyses were performed using MLWiN, version 2.30 (Centre for Multilevel Modelling, University of Bristol, Bristol, UK). Multilevel modelling was used to take clustering of baseline and follow-up measurements of preschool children in kindergarten classes within kindergartens within countries into account. Five levels were used: time, preschooler, kindergarten class, kindergarten, and country. To get more insight in the subcomponents, multilevel repeated measures analyses were also performed for the sub-items of each subcomponent.

A three-way interaction between time, group, and country was performed and significant differences by country were found. Therefore, intervention effects for total diet quality and the four subcomponents were tested in the total sample and by country, using four levels (i.e., time, preschooler, kindergarten class, and kindergarten). The analyses were adjusted for age, sex, and SES. The significance level was set at *p* < 0.05.

In order to study differences in the intervention effects on total diet quality and its four subcomponents by SES, moderation analyses were also performed using MLWiN by adding a three-way-interaction to the regression analysis. Again, the significance level was set at *p* < 0.05.

## 3. Results

### 3.1. Population Characteristics

Table 2 presents the total and country-specific population characteristics. The total sample included 4968 preschoolers (mean age 4.7 ± 0.4 years, 51.4% boys), 38.5% had a mother with a lower level of education (≤14 years of education).

### 3.2. Intervention Effects on Total Diet Quality, the Four Subcomponents, and Their Sub-Items

#### 3.2.1. Total Sample

Table 3 shows the results obtained from the multilevel repeated measures analyses for the total diet quality, the four subcomponents, and the sub-items of each subcomponent in the total sample. No significant intervention effect was found for total diet quality. For two subcomponents of total diet quality (i.e., dietary quality and dietary equilibrium), a significant intervention effect was found. In both the intervention and control group, the dietary quality increased (*p* < 0.001), with a larger increase in the intervention group (mean difference: +3.4%) compared to the control group (mean difference: +1.5%). Also, for dietary equilibrium, a small increase was found in both the intervention and the control group (*p* = 0.015), with a larger increase in the intervention group (mean difference: +0.9%) compared to the control group (+0.2%). For the subcomponents dietary diversity and the meal index, no significant intervention effect was found.

For most sub-items of each sub component, no significant intervention effect was found, except for equilibrium of fruit and snacks (*p*_equilibrium_fruit_ = 0.023; *p*_equilibrium_snacks_ = 0.018), with an increase of the equilibrium score in the intervention group (mean difference for fruit: +1.4%; mean difference for snacks: +1.8%) and a decrease in the control group (mean difference for fruit: −0.6%, mean difference for snacks: −1.6%).

#### 3.2.2. Stratified Analyses by Country

Results obtained from the multilevel repeated measures analyses for total diet quality and the four subcomponents in the six country-specific samples are shown in Table 4 and Table 5. The stratified analyses by country showed similar results as for the total sample. For the subcomponent dietary quality, a significant intervention effect was found in German and Greek preschoolers. In the German sample, an increase of dietary quality was found in the intervention group (mean difference: +3.5%), while a decrease was seen in the control group (mean difference: −0.2%) (*p* = 0.001). For the Greek sample, both in the intervention and control group an increase of dietary quality was found (*p* = 0.005), with a larger increase in the intervention group (mean difference: +5.6%) compared to the control group (mean difference: +2.5%). For dietary equilibrium, only in Belgian preschoolers a significant intervention effect was found (*p* = 0.002), with an increase of dietary equilibrium in the intervention group (mean difference: +1.1%) and a decrease in the control group (mean difference: −1.2%). For dietary diversity, the meal index, and total diet quality, no significant intervention effects were found.

### 3.3. Moderation Analyses Results

Table 6 presents the results of the moderation analyses for the total sample. SES was not a significant moderator for intervention effects on total diet quality, nor for the four subcomponents. Also, for the country-specific analyses, no significant moderation of intervention effects by SES was found.

## 4. Discussion

The aim of this study was to investigate the effect of the multibehavioural ToyBox-intervention on total diet quality and its four subcomponents in European preschoolers and to investigate if the potential intervention effects differed by SES. No intervention effects were found on total diet quality. However, for two subcomponents, promising intervention effects were found. The intervention led to a larger increase of scores on both dietary quality and dietary equilibrium in preschoolers of the intervention group compared to the control group, with a more explicit increase in dietary quality. In other words, preschoolers of the intervention group made larger improvements in making healthy choices within the different food groups than preschoolers of the control group. Since preschoolers scored particularly low on this subcomponent at baseline [13], it is a promising finding that a multibehavioural intervention can increase preschoolers’ scores on dietary quality, leading to healthier overall food choices. These results indicate that preschoolers of the intervention group had an overall better quality of food choices than their peers of the control group after implementing the intervention by, for instance, consuming more wholegrain products and plain water instead of white bread and sugared beverages. The ToyBox-intervention focused on improving the quality of beverage choices (for instance, choosing water instead of sugared beverages) and the quality of snacks (choosing healthy snacks instead of snacks high in sugar and fat). It is possible that the messages on the quality of these specific food choices made parents more aware of overall dietary quality of all food choices they provide to their preschool children, resulting in a better overall dietary quality of food choices.

For the subcomponent dietary equilibrium, preschoolers of the intervention group made larger improvements corresponding to the food-based recommendations than their peers of the control group. This is a positive finding, since these results indicate that preschoolers of the intervention group, for instance, better reached fruit and vegetable recommendations and exceeded norms of unhealthy foods such as snacks less than preschoolers of the control group after the intervention was implemented, leading to a better intake of different nutrients which can result in better health. The ToyBox-intervention not only focused on the quality of choices, but also on increasing the quantity of plain water and healthy snack consumption and decreasing soft drink and unhealthy snack consumption, referring to the recommendations. This focus on recommendations may have resulted in parents taking recommendations for all food items more into account. Although small differences, in the context of preschool children, these represent meaningful outcomes. These small improvements in dietary quality at preschool age may be very relevant if sustained or magnified through tracking into later childhood.

Unlike the promising findings for dietary quality and dietary equilibrium, no significant intervention effects were found for dietary diversity and the meal index. Already at the start of the intervention, preschoolers scored high on the meal index, meaning that many already consumed breakfast, lunch, and dinner every day. Despite the low scores on the subcomponent dietary diversity at baseline, the intervention did not lead to an increase in scores on dietary diversity. In other words, preschoolers of the intervention group did not have a significantly larger improvement of their intake of at least one food item of each food group (such as fruit, vegetables, milk, bread) every day compared preschoolers of the control group.

More detailed analyses showed no significant intervention effect on the sub-items of each subcomponent, except for equilibrium of fruit and snacks. Therefore, it can be concluded that the effect on the subcomponents dietary quality and dietary equilibrium is not only the consequence of an effect on the specific items on which the intervention focused (i.e., beverages and healthy snacks), but of the sum of a number of small (non-significant) effects on multiple sub-items. This study indicates that multibehavioural interventions with a focus on specific energy balance-related behaviours in preschoolers not only affect those targeted behaviours, but can also have more generalized effects. This may be due to an increase of parents’/caregivers’ awareness for an overall healthy lifestyle. Other intervention studies should also look into the effect of multibehavioural interventions on diet quality in order to confirm these results and find explanatory factors for these results.

No clear explanation could be found for the different intervention effects in different countries. A possible explanation for the positive intervention effect on diet quality in Polish preschoolers could be that this country-specific sample had a very low score on diet quality at baseline, the lowest score compared to the other country-specific samples, so there was more room for improvement after the intervention was implemented. Also, no similarities were found between the level of implementation of a country and intervention effects on diet quality by country [23]. Furthermore, intervention effects also depend on possible other or previous interventions that were run in the same population. If previous interventions already focused on some of the parameters included in our intervention then the chances of finding an effect are lower as the behaviour of those sensitive to interventions was probably already influenced by the previous intervention. Ongoing and previous interventions may largely differ between countries.

The second aim of this study was to investigate differences in the intervention effects based on SES. At the start of the intervention, preschoolers of lower SES backgrounds had lower scores on total diet quality and all four subcomponents compared to high SES preschool children [13]. In addition, the intervention was implemented in kindergartens, an important setting to reach lower SES preschool children because of the potential to reach all preschoolers, including those with lower SES backgrounds [36]. Interventions targeting environmental changes (such as installing water stations in kindergarten classes) can have better results in reaching lower SES people compared to interventions only targeting individual attitude, knowledge, or skills, which require more efforts and resources from the individual [37]. Based on both arguments, the lower baseline scores and the environmental component in the intervention, it could be expected that the intervention would lead to more improvement in scores on total diet quality and all four subcomponents in lower SES preschoolers compared to their high SES peers. However, the intervention effects did not differ by SES. A possible explanation for the lack of higher impact on lower SES preschoolers could be found in the fact that the ToyBox-intervention did not specifically target lower SES parents/caregivers. Lower SES parents/caregivers might have encountered difficulties in understanding the messages that preschoolers brought home as part of the educational component of the intervention, for instance, because of a lack of reading skills, since educational level of the mother was used as a SES indicator. Previous literature indicated that lower SES parents are more difficult to reach with health messages and often take health less into account than high SES parents [38,39].

A review of Moore and colleagues indicated that school-based interventions may narrow, widen, or have no effect on inequality [26]. The ToyBox-intervention did not narrow inequality in diet quality, but it also did not widen inequality, which is a promising finding. A review of McGill and colleagues on the socioeconomic inequalities in impact of interventions to promote healthy eating concluded that interventions focusing on fiscal measures, such as taxes or subsidies, appeared to decrease inequalities, and interventions focusing on individual-based information and education, such as dietary counselling, appeared to increase inequalities [40]. However, in both reviews, it was also concluded that most studies did not explore differential effects by socioeconomic status, which should be targeted in future studies [26,40]. Preventing the development of socioeconomic inequalities in unhealthy behaviours early in life is important to tackle socioeconomic inequalities in health. Therefore, future studies should further investigate differences in intervention effects by SES.

### 4.1. Future Studies

The DQI can be used in future studies to investigate overall diet quality of preschoolers, next to specific dietary behaviours. To the best of our knowledge, this was the first study that looked into the effects of a multibehavioural cluster randomized controlled trial with a focus on specific energy balance-related behaviours on total diet quality and thus more generalized effects in European preschoolers. Since promising results were found, future studies should further investigate if the current findings can be confirmed by other intervention research.

Future studies should also investigate differences in intervention effects by SES. Equity-specific subgroup analyses contribute to needed knowledge about what may work to reduce socioeconomic inequalities in obesity and underlying health behaviours [41].

### 4.2. Strengths and Limitations

The present study holds several strengths. To our knowledge, this is the first study that examined more holistic effects of an intervention focusing on specific health behaviours in European preschoolers. Another strength of this study was the large sample of preschoolers from six European countries and the cluster randomized pre- and post-test design including an intervention and control group.

There are also a number of limitations to the study. The data are self-reported, which may result in social desirability. However, this was partially covered by ensuring anonymity and because of the use of a within design and misreporting being comparable over different time moments, this is not a real problem. The FFQ used in the ToyBox-study was developed based on a previously validated FFQ developed by Huybrechts and colleagues which was specifically designed for use by parents of preschool children [30]. However, it did not specifically target children who spend time in childcare. Nonetheless, the FFQ of Huybrechts and colleagues has already been validated using a sample of children collected through kindergartens and the results showed moderate to good reproducibility and moderate to good relative validity [30]. Also, we acknowledge that the ToyBox-sample is not a fully representative European sample, due to sampling in specific regions in each country. Samples included preschoolers of low, medium, and high SES backgrounds, and in each kindergarten (almost) complete classes were included. The samples can give a fair approximation of the average situation in each country. The procedure of sampling in specific regions has also been used in several other European studies such as HELENA and ENERGY [42,43]. Socioeconomic status was only assessed by the educational level of the mother. Educational level has been identified as an important indicator for SES and it is one of the most frequently used indicators in studies on dietary intake in children [33,44]. It can be easily measured in a self-administered questionnaire such as the Primary Caregivers’ Questionnaire of the ToyBox-study [45] and also leads to a higher response rate than, for instance, income, which is often seen as contentious [46]. In addition, educational level is relevant to people regardless of age or working circumstances [45]. Nevertheless, different indicators measure different aspects of SES [47]. So, future studies should use different SES indicators and study differences on intervention effects based on different indicators.

## 5. Conclusions

The multibehavioural ToyBox lifestyle intervention led to a larger increase of scores on both dietary quality and dietary equilibrium in preschoolers of the intervention group compared to the control group, with a more explicit increase in dietary quality. Since preschoolers scored particularly low on this subcomponent, it is a promising finding that a multibehavioural intervention can increase preschoolers’ dietary quality scores, leading to healthier overall food choices. This study indicates that multibehavioural interventions with a focus on specific energy balance-related behaviours in preschoolers not only affect those targeted behaviours, but can also have more generalized effects. The ToyBox-intervention effects were similar for both lower and high SES preschoolers, so the intervention does not widen social inequality in health, as earlier intervention studies did, which is an encouraging finding for future public health interventions including all different layers of the population.

## Figures and Tables

**Table 1:** Calculation of Diet Quality Index (DQI) and subcomponents and sub-items included in analysis on item level.

**Dietary Diversity**
For each food group (*water*, *cereals*, *potatoes*, *fruit*, *vegetables*, *milk products*, *cheese, and meat and fish*):
sum frequency food items belonging to that food group
If sum ≥ 1: diversity score for particular food group = 1
If not: diversity score for particular food group = 0
Total dietary diversity = Sum diversity scores of all food groups/number of food groups (8) × 100%
**Dietary Quality**
Each food item of the food frequency questionnaire has been scored ‘−1’, ‘0’, or ‘1’ depending on whether the food item of a certain food group was categorized in:
the low-nutritious but energy-dense group (−1)
the intermediate group (0)
the preference group (1)
of the Active Flemish food triangle.
Dietary quality score for each food item = score of each food item × portion of each food item (grams/millilitres)
Total dietary quality = sum food item dietary diversity scores/total amount of all food items (grams/millilitres per day) × 100%
**Dietary Equilibrium**
For each food group: Sum total daily intake of all food items of that food group, then:
For adequacy
If daily intake food group met minimum norm: adequacy food group = 1
If not: adequacy food group = daily intake of the food group/minimum norm of the food group.
For moderation
If maximum norm food group was not exceeded: moderation food group = 0
If maximum norm food group was exceeded: moderation food group = (maximum norm − daily intake of the food group)/maximum norm
If daily intake of food group was twice as many or more than the recommended maximum intake: moderation = −1.
Equilibrium specific food group = sum adequacy and moderation specific food group
Total equilibrium score = sum equilibrium scores of all food groups/total amount of food groups (9 ^1^) × 100%
**Meal Index** = (Frequency breakfast/week + lunch/week + dinner/week)/3 × 100%
**Total DQI** = Sum subcomponents/4

^1^ To determine the equilibrium score, an extra food group is added, more specifically snacks.

**Table 2:** Baseline characteristics of participants of the total sample and each country separately.

	Total	Belgium	Bulgaria	Germany	Greece	Poland	Spain
*N*	4968	769	644	882	825	1021	827
Age (years)	4.7 ± 0.4	4.4 ± 0.5	4.9 ± 0.3	4.5 ± 0.5	4.9 ± 0.3	4.9 ± 0.3	4.9 ± 0.3
Gender (% male)	51.4	50.7	49.8	51.8	50.4	50.9	54.5
SES, % lower SES * (=% ≤14 years of education)	38.5	34.1	40.5	51.4	52.9	20.7	34.8

* (Years of school education mother). SES, socioeconomic status.

**Table 3:** Intervention effects for total diet quality, the four subcomponents and all sub-items in the total sample (adjusted for age, sex, SES).

	Time × Condition β	Group	Mean Baseline	Mean Follow-Up
Dietary Diversity	−0.34	I	64.6	63.8
		C	63.7	63.3
Diversity Bread	−0.02			
Diversity Potatoes	−0.03			
Diversity Fruit	0.01			
Diversity Vegetables	−0.02			
Diversity Milk	−0.00			
Diversity Cheese	0.02			
Diversity Meat	0.01			
Dietary Quality	1.97 ***	I	58.5	61.9
		C	59.1	60.6
Quality Water	19.92			
Quality Bread	−1.09			
Quality Potatoes	−0.68			
Quality Fruit	−0.46			
Quality Milk	0.88			
Quality Meat	−1.11			
Dietary Equilibrium	0.82 **	I	66.8	67.7
		C	66.6	66.8
Equilibrium Water	0.01			
Equilibrium Bread	−0.00			
Equilibrium Potatoes	−0.01			
Equilibrium Fruit	0.02 *			
Equilibrium Vegetables	0.01			
Equilibrium Milk	−0.01			
Equilibrium Cheese	0.00			
Equilibrium Meat	0.00			
Equilibrium Snacks	0.03 *			
Meal Index	−0.40	I	91.1	89.3
		C	90.0	88.6
Breakfast	0.00			
Lunch	−0.01			
Dinner	−0.01			
Total Diet Quality	0.52	I	70.3	70.7
		C	69.8	69.8

* *p* < 0.05; ** *p* < 0.01; *** *p* < 0.001; I = intervention group; C = control group.

**Table 4:** Intervention effects for total diet quality and the four subcomponents in Belgium, Bulgaria, and Germany (adjusted for age, sex, and SES).

	Group	Belgium			Bulgaria			Germany		
		Mean Baseline	Mean Follow-Up	Time × Condition β	Mean Baseline	Mean Follow-Up	Time × Condition β	Mean Baseline	Mean Follow-Up	Time × Condition β
Dietary Diversity	I	72.7	69.8	−1.21	59.8	60.3	0.80	64.5	64.2	−1.29
	C	72.5	70.8		60.0	59.7		65.1	66.0	
Dietary Quality	I	59.5	63.0	2.14	63.5	64.2	−0.90	63.3	66.8	3.72 **
	C	58.7	60.0		65.4	67.0		63.9	63.7	
Dietary Equilibrium	I	68.3	69.4	2.36 **	63.0	65.4	0.75	69.0	69.7	0.30
	C	69.0	67.8		63.0	64.6		68.2	68.7	
Meal Index	I	95.1	94.3	−0.14	94.4	91.7	−5.66	94.5	93.9	2.54
	C	95.6	94.9		90.9	93.8		95.7	92.6	
Total Diet quality	I	73.8	74.0	0.79	70.2	70.4	−1.25	72.8	73.6	1.32
	C	73.9	73.3		69.9	71.3		73.2	72.7	

** *p* < 0.01; I = intervention group; C = control group.

**Table 5:** Intervention effects for total diet quality and the four subcomponents in Greece, Poland, and Spain (adjusted for age, sex, and SES).

	Group	Greece			Poland			Spain		
		Mean Baseline	Mean Follow-Up	Time × Condition β	Mean Baseline	Mean Follow-Up	Time × Condition β	Mean Baseline	Mean Follow-Up	Time × Condition β
Dietary Diversity	I	62.7	61.5	1.18	63.6	63.4	−0.88	64.6	63.8	0.12
	C	62.6	60.3		61.2	61.9		61.8	60.8	
Dietary Quality	I	57.2	61.0	1.31	48.5	54.1	3.16 **	59.9	62.2	1.03
	C	58.8	61.4		48.9	51.4		59.8	61.0	
Dietary Equilibrium	I	68.6	69.1	0.79	66.7	67.0	0.25	64.7	65.7	0.56
	C	69.3	69.0		65.8	65.9		64.5	65.0	
Meal Index	I	78.5	74.7	−0.52	95.9	95.0	−0.91	87.8	85.9	1.55
	C	74.0	70.7		95.4	95.4		86.7	83.3	
Total Diet quality	I	66.7	66.6	0.69	68.7	69.9	0.41	69.2	69.4	0.82
	C	66.2	65.3		67.8	68.7		68.1	67.5	

** *p* < 0.01; I = intervention group; C = control group.

**Table 6:** Moderation analyses on the total diet quality and the four subcomponents in the total sample with SES as possible moderator for the intervention effect (adjusted for age and sex).

	Time × Condition × SES β	*p*
Dietary Diversity	0.63	0.343
Dietary Quality	0.36	0.516
Dietary Equilibrium	−0.21	0.585
Meal Index	1.51	0.173
Total Diet quality	0.56	0.147
